# Some recollections about kuru in a patient at Rabaul in 1978, and subsequent experiences with prion diseases

**DOI:** 10.1098/rstb.2008.4029

**Published:** 2008-11-27

**Authors:** Euan M. Scrimgeour

**Affiliations:** Infectious and Tropical Diseases51 Woodroyd Street, Mount Lawley, WA 6050, Australia

## 1. Kuru patient: clinical course and Autopsy

I first heard about kuru when I was undertaking the six-month full-time Diploma in Tropical Medicine and Hygiene (DTM&H) course at Edinburgh University in 1970–1971. The Head of the Department of Infectious and Tropical Diseases, the eminent Dr Frederick J. Wright, had visited Papua New Guinea and, as was customary for this superb physician and teacher, was able to give the class a first-hand account of a kuru patient. His example and inspiration later led to the establishment of the DTM&H course in the Sultanate of Oman (Scrimgeour 2004). In the latter course, kuru and its importance in prion disease epidemiology were again mentioned.

In 1978, in Rabaul, East New Britain, a patient from the Mission Hospital at Vunapope was referred to me, as the regional consultant physician, with a tentative diagnosis of cerebellar dysfunction. There was a suggestion that alcohol might have been a contributing factor. The patient was not a Tolai, the major ethnic group in East New Britain, but a highlander from the Fore region of the Eastern Highlands Province. The coconut and cocoa plantations in East New Britain employed large numbers of highlanders, and the patient had been noted to stumble in the plantations and, as his gait deteriorated, he was unable to continue working.

**Figure 1:**
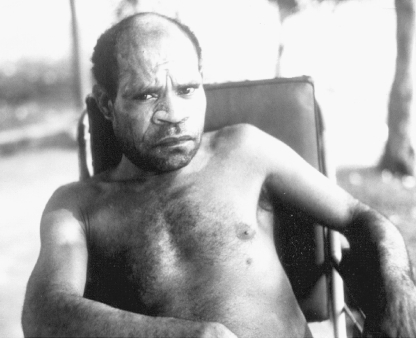
Kuru patient from the Fore region of the Eastern Highlands Province, 1978.

On examination, he was a pleasant but apprehensive middle-aged highlander, who spoke fluent Tok Pisin (Melanesian Pidgin; figure 1). He gave a history of several months' progressive, marked gait disturbance and denied alcohol consumption. Clinical examination revealed cerebellar dysfunction with severe ataxia, and since he came from the Fore region, kuru was suspected. Routine haematology, biochemistry and microbiological investigations, and plain radiology of the skull and brain, were normal. Further investigation was not available in Rabaul. When the findings and provisional diagnosis were discussed with the patient, he became very distressed, and said he had suspected for some time that he had kuru. He recalled being present as a small child when brains from kuru patients were consumed in his village, and he was familiar with the signs and symptoms. Repatriation to Okapa was offered, but he declined, stating that he was fearful of returning to his community, where there were ‘bad spirits’. Some of his friends from Okapa who were working with him tried to persuade him to return, but he was adamant that he would not go back to the highlands. Thus, for the ensuing four months, he remained an inpatient in the Base Hospital, and it was possible to document his steady deterioration day by day and to obtain a comprehensive neurological record of his illness on cine films and slides. Eventually, he developed advanced dementia, became bedfast, mute and, finally, terminally ill.

A decision had been made to conduct an autopsy when he died, and to preserve the brain in dry ice for subsequent evaluation in Dr D. Carleton Gajdusek's Laboratory of Central Nervous System Studies in the National Institutes of Health (NIH), Bethesda, Maryland. I had never conducted an autopsy, and the procedure was planned with great care. Two medical registrars were invited to help, but only the Papua New Guinean Registrar, Dr Joseph Kaven, agreed. When the patient was finally pronounced dead, within an hour the autopsy began. Both operators wore full theatre dress, with visors and safety glasses, and were double-gloved. There was no air conditioning in the mortuary and the temperature and tropical humidity were very high; within a few minutes, both operators were perspiring profusely, the glasses became misted and the theatre gowns became drenched. The procedure was conducted slowly and with great care to avoid sharps injury or splashing. The skull was opened with a hand-saw, and the brain was removed and placed in dry ice. No untoward event occurred, and no splashing was noted, but at the conclusion of the autopsy, an observer (also gowned and gloved, and wearing safety glasses) at the back of the mortuary, who was taking photographs, found a few drops of blood on the camera. Since she was standing approximately 3 m from the mortuary table, and no blood was seen on the tiled floor, this was hard to explain. At the conclusion of the autopsy, all theatre equipment, including safety glasses and theatre shoes, and all instruments were double-bagged and taken for incineration, and terminal cleaning of the mortuary proceeded with extra care. The patient's body was incinerated.

The brain preserved in dry ice was collected by Dr Michael Alpers, Director of the Papua New Guinea Institute of Medical Research and transported to the NIH, where subsequent tests confirmed kuru, and led to successful transmission experiments (Scrimgeour *et al.* 1983).

## 2. Further experience of prion diseases

In subsequent years, experience of kuru led to further opportunities for working with prion diseases. In 1986, I spent a year in the NIH, and was part of the team investigating Creutzfeldt–Jakob disease (CJD) and other prion diseases. Later, in 1991, while working in public health in Glasgow, Scotland as the bovine spongiform encephalopathy (BSE) and variant CJD epidemic unfolded, I visited an abattoir near Glasgow to observe the procedure for slaughter of cattle. It had been stipulated at this time that the brain and spinal cord of animals should not enter the food chain. To extract brain from cattle, slaughtermen, having removed the head of animals exposed to BSE, inserted a high-pressure air hose into the foramen magnum and blew the brains out through the orbital fissures before discarding the head. A shower of tissue and blood splattered around. The workers carried out this manoeuvre without visors or gloves, and casually ate their sandwiches between processing heads. I observed these operations in disbelief from the rear of the room.

A letter describing this practice and warning of the potential hazard (Scrimgeour & Brown 1991) later led to new government regulations for processing neurological tissue from cattle exposed to BSE. A further letter to *Veterinary Record* commented on the discarding of partly processed specified offal (tissues potentially contaminated with prions) from cattle possibly exposed to BSE in landfill areas near Glasgow, where birds and feral animals could forage and conceivably transfer prions elsewhere (Scrimgeour *et al.* 1996**). New procedures were introduced to sterilize specified offal adequately before being discarded.

Finally, in 1996, while working in the Sultanate of Oman, I encountered two patients with sporadic CJD, the first to be diagnosed in Oman (Scrimgeour *et al.* 1996**). On these occasions, I carried out lumbar punctures with stringent precautions to obtain diagnostic cerebrospinal fluid to be sent to the NIH. Oman now banned the importation of beef from the UK, and we formed a variant CJD Surveillance Committee. Until now, no cases of variant CJD have been documented in Oman but since large amounts of British beef, including sausages, had been imported in the preceding decade, the potential risk of future cases remains.

Thus, from the early encounter with the Rabaul case, and encounters with several other cases of kuru in the highlands in the late 1970s, these experiences led to many opportunities to work with prion diseases. It is interesting to contemplate that there must be few individuals who have carried out only one autopsy during their careers, and even fewer whose subject died of kuru.
